# Supplementary material for: Identification of photoperiod-induced specific miRNAs in the adrenal glands of Sunite sheep (Ovis aries)
Source: Front Vet Sci. 2022 Jul 22;9:888207. doi: 10.3389/fvets.2022.888207 (PMC9354845; doi:10.3389/fvets.2022.888207)
Supplement: Supplementary file 1 [file Data_Sheet_1.ZIP › Supplementary file/miRDeep2 file.zip!/miRDeep2 file/Know_Structure/SPLP42A3/oar-mir-485.pdf]

miRBase precursor : oar-mir-485
Total read count : 1096
oar-miR-485-5p read count : 524
oar-miR-485-3p read count : 571
remaining reads : 1

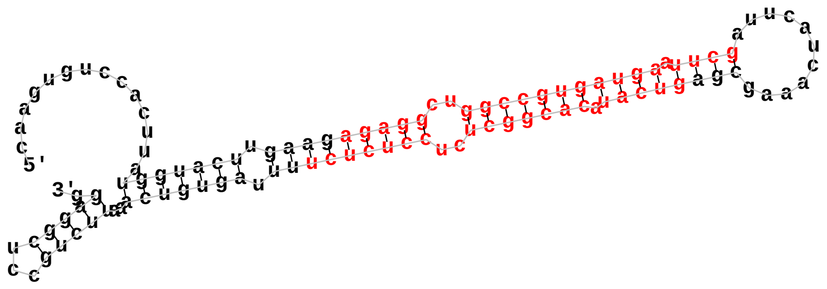

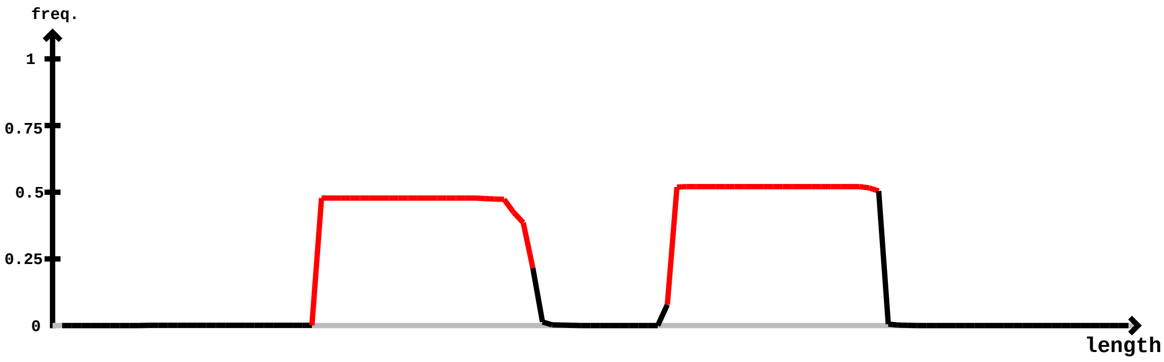


```
                                oar-miR-485-5p
                                                            oar-miR-485-3p
5'- caaguguccacuuaugguacuugaagagaggcuggccgugaugaauucgauucaucaaagcgagucauacacggcucuccucucuuuuagugucaaauucugccucggagg   -3'    exp
    ((((((.(((....)))))))))((((((((..((((((((((.((((...........)))))))).)))))))..))))))))..........(((((...))))).    reads   mm   sample
    ........cacuuaugguacuugaag.....................................................................................    1       0    seq
    ..........................agaggcuggccgugaug....................................................................    2       0    seq
    ..........................agaggcuggccgugauga...................................................................    2       0    seq
    ..........................agaggcuggccgugaugaa..................................................................    1       0    seq
    ..........................agaggcuggccgugaugaau.................................................................    54      0    seq
    ..........................agaggcuggccgugaugaauu................................................................    42      0    seq
    ..........................agaggcuggccgugaugaauuc...............................................................    187     0    seq
    ..........................agaggcuggccgugaugaauucg..............................................................    221     0    seq
    ..........................agaggcuggccgugaugaauucga.............................................................    12      0    seq
    ..........................agaggcuggccgugaugaauucgau............................................................    1       0    seq
    ..........................agaggcuggccgugaugaauucgauu...........................................................    1       0    seq
    ..........................agaggcuggccgugaugaauucgauuc..........................................................    1       0    seq
    ..............................................................agucauacacggcucuccucu............................    1       0    seq
    ..............................................................agucauacacggcucuccucuc...........................    1       0    seq
    ..............................................................agucauacacggcucuccucucu..........................    84      0    seq
    ...............................................................gucauacacggcucuccucu............................    4       0    seq
    ...............................................................gucauacacggcucuccucuc...........................    12      0    seq
    ...............................................................gucauacacggcucuccucucu..........................    461     0    seq
    ...............................................................gucauacacggcucuccucucuu.........................    4       0    seq
    ...............................................................gucauacacggcucuccucucuuu........................    1       0    seq
    ...............................................................gucauacacggcucuccucucuuuu.......................    1       0    seq
    ................................................................ucauacacggcucuccucucu..........................    2       0    seq
```
